# Copine 3 “CPNE3” is a novel regulator for insulin secretion and glucose uptake in pancreatic β-cells

**DOI:** 10.1038/s41598-021-00255-0

**Published:** 2021-10-19

**Authors:** Waseem El-Huneidi, Shabana Anjum, Abdul Khader Mohammed, Hema Unnikannan, Rania Saeed, Khuloud Bajbouj, Eman Abu-Gharbieh, Jalal Taneera

**Affiliations:** 1 Department of Basic Medical Sciences, College of Medicine, University of Sharjah, Sharjah, United Arab Emirates; 2 University of Sharjah, Sharjah Institute for Medical Research, Sharjah, United Arab Emirates; 3 Department of Clinical Sciences, College of Medicine, University of Sharjah, Sharjah, United Arab Emirates

**Keywords:** Type 2 diabetes, RNAi

## Abstract

Copine 3 (*CPNE3*) is a calcium-dependent phospholipid-binding protein that has been found to play an essential role in cancer progression and stages. However, its role in pancreatic β-cell function has not been investigated. Therefore, we performed a serial of bioinformatics and functional experiments to explore the potential role of *Cpne3* on insulin secretion and β-cell function in human islets and INS-1 (832/13) cells. RNA sequencing and microarray data revealed that *CPNE3* is highly expressed in human islets compared to other *CPNE* genes. In addition, expression of *CPNE3* was inversely correlated with HbA1c and reduced in human islets from hyperglycemic donors. Silencing of *Cpne3* in INS-1 cells impaired glucose-stimulated insulin secretion (GSIS), insulin content and glucose uptake efficiency without affecting cell viability or inducing apoptosis. Moreover, mRNA and protein expression of the key regulators in glucose sensing and insulin secretion (Insulin, GLUT2, NeuroD1, and INSR) were downregulated in *Cpne3*-silenced cells. Taken together, data from the present study provides a new understanding of the role of *CPNE3* in maintaining normal β-cell function, which might contribute to developing a novel target for future management of type 2 diabetes therapy.

## Introduction

Type 2 Diabetes mellitus (T2D) is the fastest growing disorder worldwide, with an estimation to affect more than 640 million people by 2040 (http://www.diabetesatlas.org). T2D is characterized by the insufficiency of insulin secretion and/or insulin action^1^. Genetic predisposition, obesity, sedentary life, and rapid socio-economic shift have been ascribed as the leading causes of the disease's high prevalence^2,3^. Several tissues, including pancreatic β-cells, liver, muscle and adipose tissues, play a significant role in glucose metabolism and the pathophysiology of T2D^4^. Pancreatic β-cells are specialized cells that produce insulin in response to specific nutrients, primarily glucose, and amino acids and fatty acids to a lesser extent^5^. Typically, glucose is transported into the β-cell^6^, activating ATP production through aerobic respiration, leading to the closure of the KATP channel. Accumulation of K^+^ in the cytoplasm will lead to the depolarization of the membrane, followed by an influx of Ca^2+^ by the opening of the voltage-dependent Ca^2+^ channel. Consequently, this will initiate exocytosis of insulin granules. Despite increased knowledge about the pathogenesis of T2D, much remains unknown about genes involved in the disease. To date, genome-wide association studies (GWAS) have identified more than 140 common genetic variations linked to T2D and pancreatic-cell dysfunction^7,8^. However, the established genetic variants can only account for around 10% of the disease's heritability^9,10^. This emphasizes the importance of identifying candidate genes involved in insulin secretion and β-cell function.

Copines are calcium-dependent phospholipid-binding proteins encoded by *CPNE* genes that are evolutionally conserved in eukaryotic organisms with nine family members (*CPNE1*-*9*) have been identified^11^. In humans, *CPNE* genes were reported in various tissues such as the hippocampus, liver, spleen and phagocytic blood cells^12^. Recent reports have suggested that copine genes may have an essential role in cancer progression and stages^13–16^. Among the copine family, *CPNE3* gene produces a polypeptide with two types II C2 domains in the amino terminus and “A” domain–like a sequence in the carboxy terminus that mediates integrin–extracellular ligand interactions*.* CPNE3 was first identified in the ciliate protozoan Paramecium Tetraurelia^12,17,18^. *CPNE3* is involved in the pathogenesis of breast, prostate, leukemia and small cell lung cancers^12,19–23^; however, its role in pancreatic β-cell function remains unidentified. Herein, we utilized RNA-sequencing and microarray data to analyzed the expression of *CPNE* family in human pancreatic islets. Several functional experiments were performed in INS-1 (832/13) cells, including siRNA silencing, insulin secretion, glucose uptake, cell viability and apoptosis to explore the role of *Cpne3* in β-cell function.

## Results

### Expression profile of copine genes in human islets and INS1 cell line

**Figure 1:**
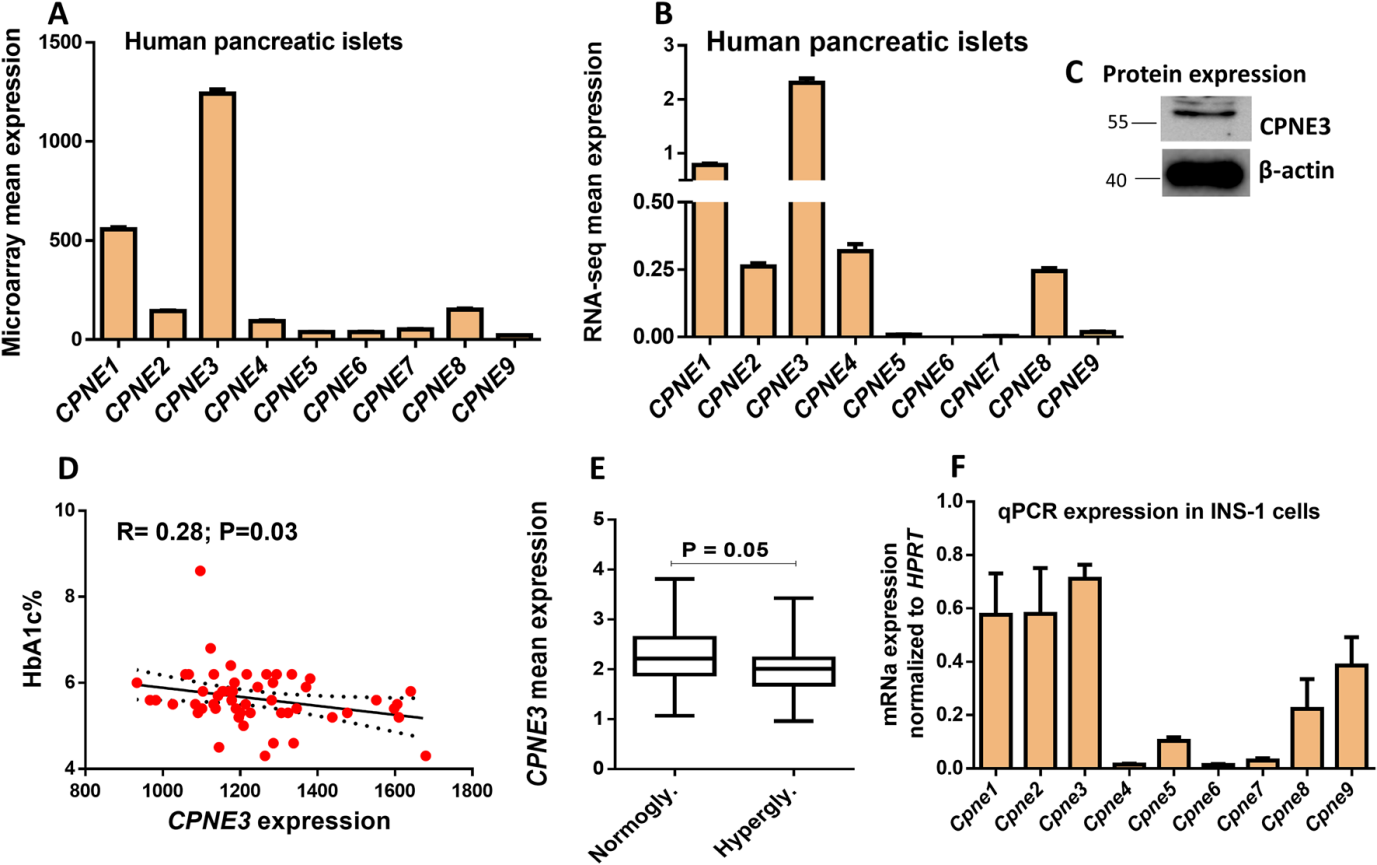
Expression profile of copine genes in human pancreatic islets. (**A**) Microarray mean expression of the nine copine genes in nondiabetic human islets (*n* = 67). (**B**) RNA sequencing expression of copine genes in nondiabetic human islets (*n* = 67). (**C**) Western blot expression of *CPNE3* obtained from nondiabetic human islets (n = 1; obtained from Prodo Lab. Inc, CA., USA). (**D**) Analysis of microarray expression correlation of *CPNE3* (*n* = 67) with HbA1c levels. R and p-values are indicated in the respective graphs. (**E**) Differential expression analysis of *CPNE3* (RNA-seq) in human islets obtained from hyperglycemic donors (*n* = 27) vs. human islets from normoglycemic donors (*n* = 50). (**F**) q-PCR expression analysis of the nine copine members in INS-1 (832/13) cells. R: correlation coefficient; p: p-value. Bars represent mean ± SEM.

Using publicly available RNA-seq and microarray gene expression data obtained from many human pancreatic islets, we mapped the expression profile of the copines family. As shown in Fig. 1A, the microarray expression of *CPNE3* was the highest expressed gene, followed by *CPNE1*, compared to other CPNE genes. To corroborate the microarray expression data, we analyzed the expression profile of CPNE genes using RNA-seq from human islets. As illustrated in Fig. 1B, *CPNE3* gene showed the highest expression compared with other *CPNE* genes. Similarly, *CPNE1* was ranked as the second-highest expressed gene (Fig. 1B). Based on these expression data in human pancreatic islets, *CPNE3* was selected for functional investigations in pancreatic β-cell function. *CPNE3* expression was further validated at protein level from fresh nondiabetic human pancreatic islets (n = 1) (Fig. 1C). Although microarray expression of *CPNE3* was correlated negatively with HbA1c levels (Fig. 1D), no differential expression of *CPNE3* in hyperglycemic donors (HbA1c ≥ 6%) compared to normoglycemic (HbA1c < 6%) donors was observed (not shown). Interestingly, RNA-seq expression for *CPNE3* in hyperglycemic donors (HbA1c ≥ 6%) was significantly reduced (p ≤ 0.05) compared to normoglycemic (HbA1c < 6%) donors (Fig. 1E). Additionally, as clonal rat INS-1 cells are the most commonly used tool for functional validation, we profiled the expression of copine genes in INS-1 cells (n = 3). As shown in Fig. 1F, qPCR expression analysis revealed that all of the copine genes were expressed in INS-1 (832/13) cells. *Cpne2* (Ct = 14.2) was the highest expressed gene based on the Ct values, whereas *Cpne3* (Ct = 22.5) was ranked the fourth.

### Silencing of *Cpne3* in INS-1 (832/13) cells influences insulin secretion

**Figure 2:**
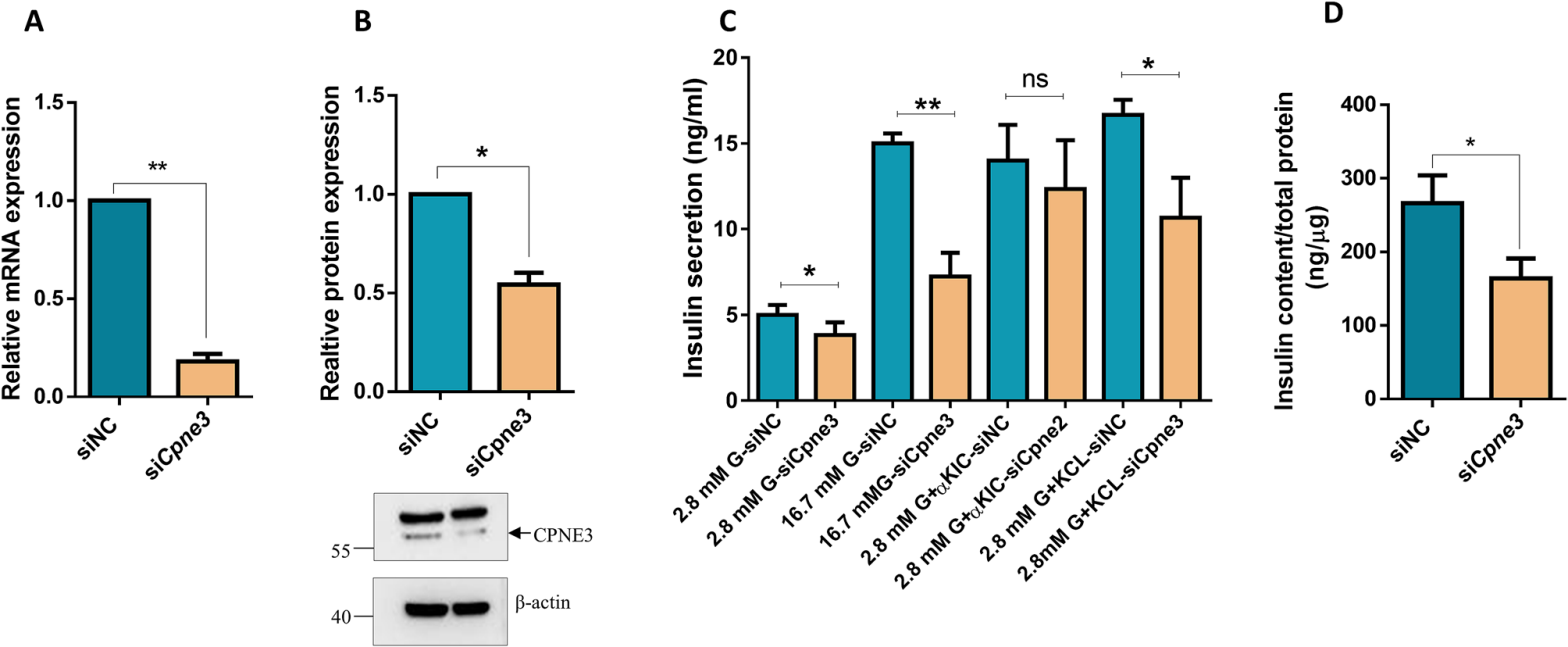
Silencing of *Cpne3* impairs insulin secretion in INS-1 (832/13) cells. (**A**) Silencing efficiency of *Cpne3* 48 h after transfection as measured by q-PCR. (**B**) Western blot expression analysis of CPNE3 in INS-1 cells transfected with siRNA against *Cpne3* or siRNA negative control relative to the endogenous control β-actin 48 h post-transfection (upper panel). Fold change in the intensity of the western blot band of CPNE3 protein expression relative to the endogenous control β-actin in *Cpne3*-silenced cells or negative siRNA control (lower panel). (**C**) Normalized stimulated insulin secretion in response to 2.8 mM glucose, 16.7 mM glucose, 10 mM α-KIC or 35 mM potassium chloride (KCl) in the presence of 2.8 mM glucose in negative control or *Cpne3*-silenced cells for one static hour of incubation. (**D**) Insulin content measurements normalized to protein content in *Cpne3*-silenced cells compared to negative control cells. Data are obtained from three independent experiments. *p < 0.05 and **p < 0.01. Bars represent mean ± SD.

As *Cpne3* was chosen for functional studies in pancreatic β-cell function, we performed siRNA-silencing of *Cpne3* in INS-1 (832/13). Silencing efficiency assessment 24 h post-transfection by qPCR exhibited a significant reduction (75%; *p* < 0.05) in mRNA expression of *Cpne3* as compared to the negative control (Fig. 2A). This was further confirmed at the protein level as evaluated by western blot analysis (Fig. 2B). To assess the impact of *Cpne3* silencing on insulin secretion, transfected cells were incubated for 1 h with 2.8 mM or 16.7 mM glucose. The results revealed a decrease in glucose-stimulated insulin secretion (GSIS) at 2.8 mM glucose (~ 20%; *p* < 0.05) and 16.7 mM glucose (~ 40%; *p* < 0.01) (Fig. 2C). More, a significant reduction (~ 40%; *p* < 0.05) on insulin secretion was observed in *Cpne3*-silenced cells stimulated with 35 mM KCl (a depolarizing agent) for 1 h compared to control cells (Fig. 2C). On the other hand, no significant effect was found when *Cpne3*-silenced cells were stimulated with 10 mM α-KIC (an agent that stimulates mitochondrial metabolism and ATP synthesis) as shown in Fig. 2C. Moreover, measurement of insulin content in *Cpne3*-silenced cells revealed a significant reduction (∼ 40; *p* < 0.05) than the negative control cells (Fig. 2D).

### Impact of *Cpne3* silencing on cell viability, cell proliferation, apoptosis and glucose uptake.

**Figure 3:**
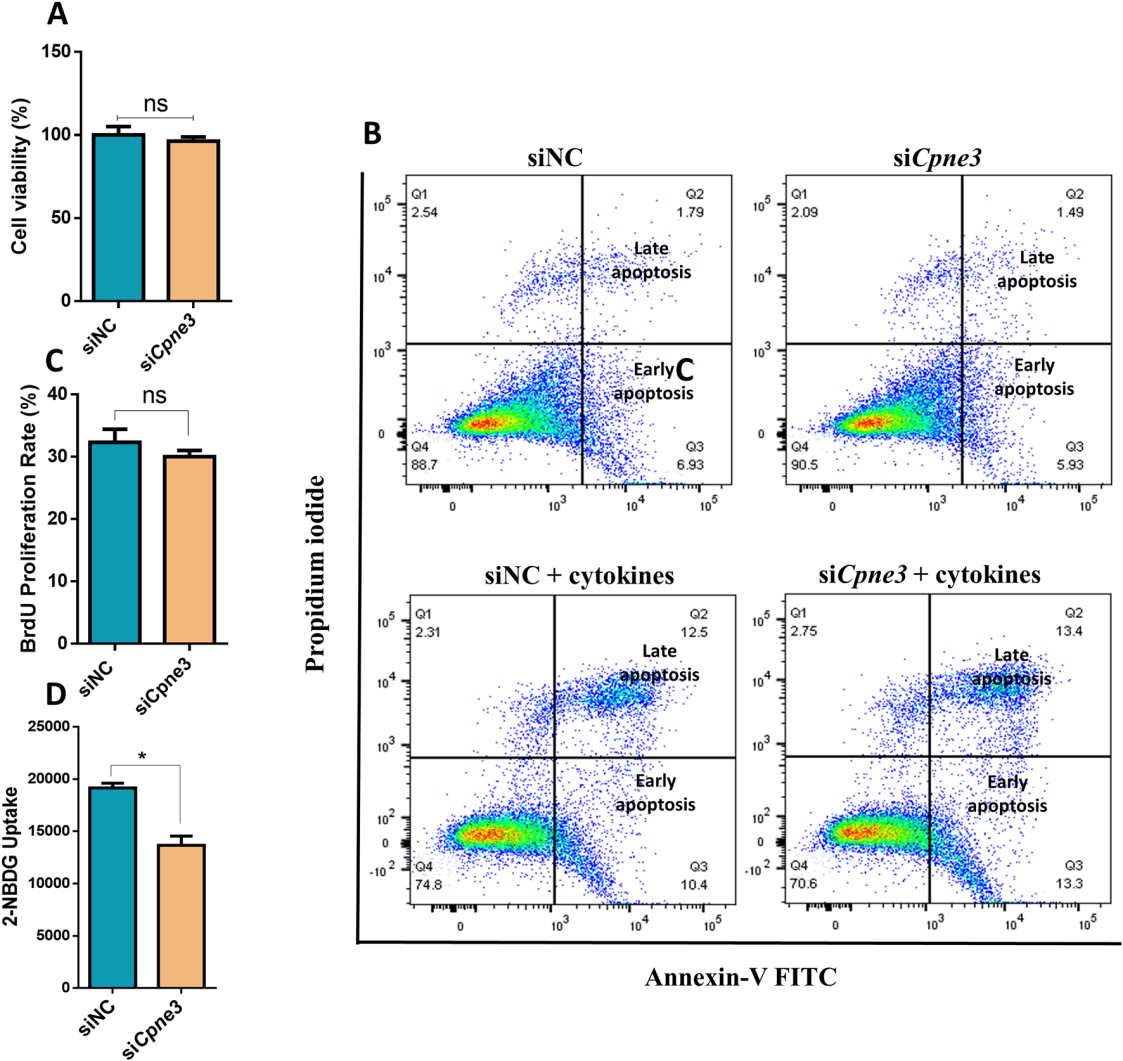
Influence of *Cpne3*-silencing on apoptosis and glucose uptake levels. (**A**) Percentage of cell viability was determined by MTT assay in *Cpne3*-silenced cells compared to control cells. (**B**) Analysis of apoptosis level in *Cpne3*-silenced INS-1 cells or negative control in the absence (upper panels) or presence (lower panels) of cytokine stimulation as analyzed by flow cytometry. (**C**) Evaluation of cell proliferation in Cpee3-silenced cells compared to control cells. (**D**) Evaluation of glucose uptake efficiency in *Cpne3*-silenced cells compared to control cells. Data are obtained from 3 independent experiments. Bars represent mean ± SD.

To gain more insights on a possible mechanistic defect of *Cpne3* silencing on insulin secretion, we tested the effect of *Cpne3* silencing on cell viability, apoptosis and glucose uptake. As shown in Fig. 3A, cell viability assessment by MTT assay showed to be unaffected in *Cpne3*-silenced cells compared to the negative control. For apoptosis analysis, Annexin-V and PI staining were used to assess apoptosis in transfected and control cells in the presence or absence of a combination of pro-apoptotic cytokines (IL-1β, TNFα and INFγ) for 24 h. As illustrated in Fig. 3B, the percentage of apoptosis (early and late) in transfected cells cultured in the absence or presence of cytokines was comparable to control cells. In addition, we investigated the impact of *Cpne3* silencing on cell proliferation using EdU labeling assay. We found that *Cpne3* silencing has no effect on cell proliferation when compared to normal cells as shown in Fig. 3C. To this end, we investigated the impact of Cpne3 silencing on glucose uptake in INS-1 (832/13) cells. As shown in Fig. 3D, a marked reduction (~ 40%) of glucose uptake was observed in *Cpne3*-silenced cells compared to control cells.

### *Cpne3* silencing influences β-cell functional genes in INS-1 (832/13)

**Figure 4:**
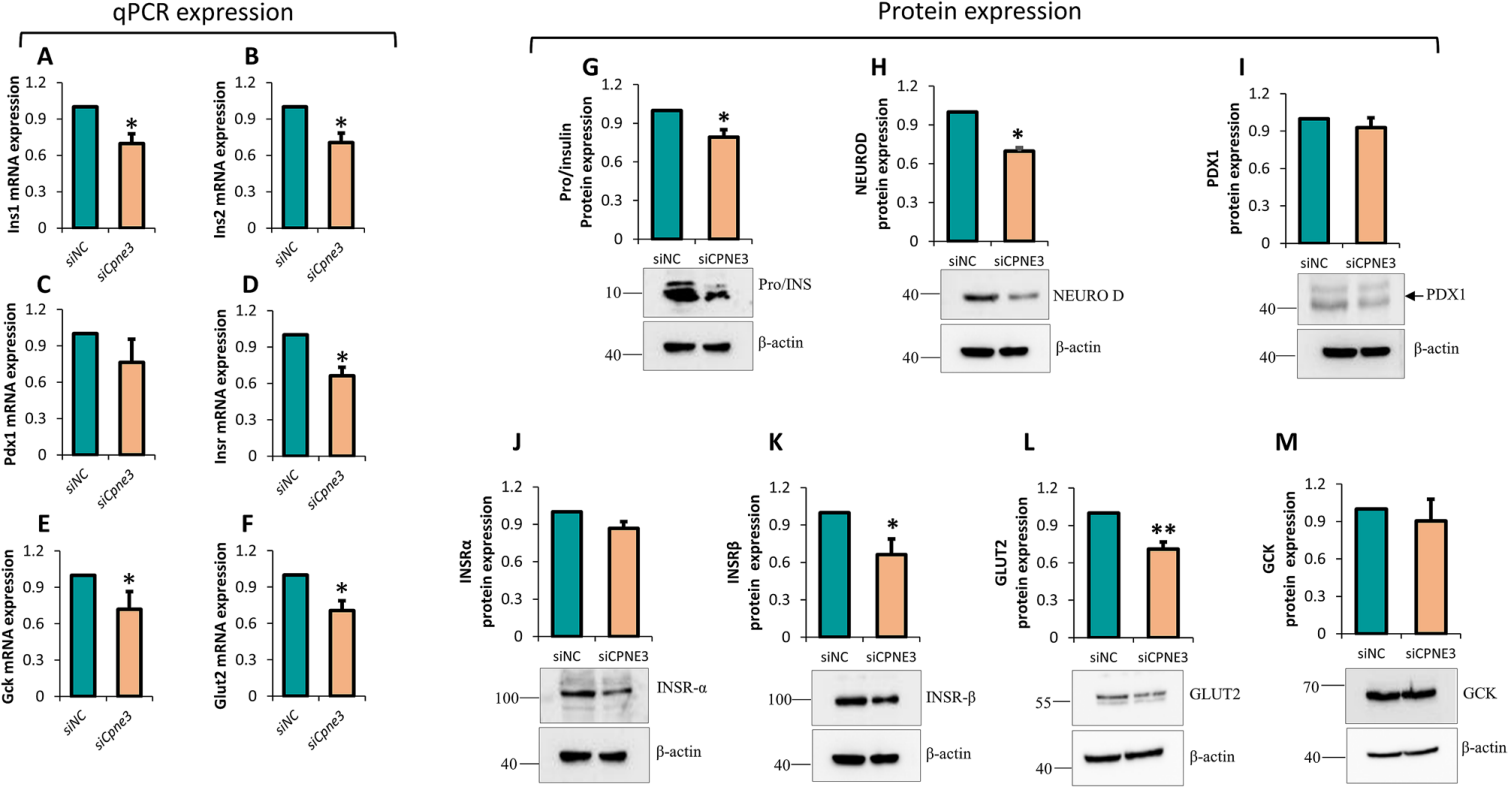
Impact of *Cpne3* silencing on β-cell function genes. Total RNA and protein materials were extracted from *Cpne3*-silenced cells or siRNA negative control 48 h post-transfection and subjected for qPCR and western blot analysis. (**A**–**F**) qPCR expression analysis of *Ins1*, *Ins2*, Pdx1, *Glut2*, *Insr* and *Gck*. (**G**–**M**) Western blot analysis of Pro/insulin, NeuroD1, PDX1, INSRα, INSβ, GLUT2 and GCK relative to the endogenous control protein β-actin. Corresponding fold change in the intensity of the western are shown below each blot. We used the same β-actin for INSRβ and PDX1 as well for GLUT2 and GCK. Data are obtained from three independent experiments except for INSRα which was run twice. p-value. *p < 0.05 and **p < 0.01. Bars represent mean ± SD.

The consequences of *Cpne3* silencing on the function of β-cell were investigated for several genes at mRNA and protein levels. Analysis of the mRNA expression of genes involved in insulin production revealed a significant (~ 30%; *p* < 0.05) downregulation of *Ins1* and *Ins2* (Fig. 4A,B). Expression of *Pdx1* was not affected in *Cpne3*-silenced cells relative to the negative control (Fig. 4C). Likewise, protein expression was markedly reduced of pro/insulin (~ 20%; *p* < 0.05) and NeuroD1 (~ 30%; *p* < 0.05) (Fig. 4G,H), whereas PDX1 expression was not affected in *CPNE3*-silenced cells relative to the negative control (Fig. 4I). More, analysis of mRNA expression of *Glut2*, *Insr* and *Gck*, which involved in glucose-sensing and insulin signaling were significantly downregulation of *Glut2* (~ 30%; *p* < 0.05)(Fig. 4F), *Insr* (~ 30%; *p* < 0.05) (Fig. 4D) and *Gck* (~ 30%; > 0.05)(Fig. 4E). Protein expression analysis further confirmed the decrease of INSRβ and GLUT2 (~ 35%; *p* < 0.05), respectively (Fig. 4K,L). GCK and INSRα were not affected (Fig. 4J,M) in *Cpne3*-silenced cells relative to the negative control.

## Discussion

In this study, we profiled the transcript levels of the 9 members of copine family in human pancreatic islets using microarray and RNA-sequencing data obtained from a large number of donors. *CPNE3* was highly expressed in human islets, inversely correlated with HbA1c levels and reduced in hyperglycemic islets. In vitro, functional studies showed that the silencing of *Cpne3* in INS-1 cells impaired insulin secretion reduced glucose uptake efficiency and downregulated expression of *Ins1*, *Ins2*, *Insr* and *Glut2*. Taken together, the provided bioinformatics and functional evidence suggest that *CPNE3* is an important regulator for pancreatic β-cell function.

This study is the first to link *CPNE3* with the pathophysiology of diabetes to the best of our knowledge. Previously, *CPNE3* was linked with cancer pathogenesis and metastasis; for example, CPNE3 was shown to up-regulate in breast tumors and glioblastoma^24^ and regulate ErbB2-dependent cancer cell motility in breast cancer^19^. Additionally, *CPNE3* was reported to be downregulated in patients with acute myocardial infarction^25^. The finding that *CPNE3* was highly expressed in human islets among other *CPNE* genes, while *Cpne2* was the highest in rat INS-1 cells is not surprising.

Such expression disparity could be attributed to a homogenous expression pattern within all islet cells or being highly expressed by different cell population in pancreatic islets such as β, α or δ cells as reported on the heterogeneity of islet-cell where certain proteins are expressed in a specific sub-population of β-cells^26^. Also, the variations in the expression pattern of copine genes may ascribed to species-specificity (human vs. rat) or the nature of investigated cells that there are differences between freshly isolated normal cells and immortalized cells^27,28^. Importantly, understanding the difference of species expression specificity is of great importance for selecting the precise validation functional model.

Expression analysis showed that *CPNE3* is reduced in hyperglycemic islets (Fig. 1). However, the finding raises a question on whether the observed reduction is causative for T2D or a consequence of glucotoxicity. A previous study showed no effect of short-term exposure of human islets to high glucose concentrations on *CPNE3* expression^29^. Typically, expression change is considered a consequence of glucotoxicity when the change after glucose exposure has a similar direction to diabetic islets. Still, it is early to consider reducing *CPNE3* expression in diabetic islets as causative for the pathogenesis of T2D. Moreover, the fact that *CPNE3* expression is correlated negatively with HbA1c levels (which is a marker for blood sugar levels) indicates the important role of *CPNE3* in glucose metabolism.

As demonstrated, gene silencing of *Cpne3* in INS-1 cells dramatically affected insulin secretion without cytotoxic effect or apoptosis (Fig. 3). Although the expression of *CPNE3* was associated with cancer pathogenesis and metastasis^13–16,19,24^, this could indicate that *CPNE3* has a differential physiological function/role that depends on the cell or microenvironment. *Cpne3*-silenced cells exhibited a down-regulation at mRNA and protein levels of genes involved in insulin biosynthesis, *Ins1* and *Ins2* but not the transcription factors that activate the insulin gene promoter (*Pdx1*)^18–20^. Additionally, expression of *Glut2*, *Gck* and *Insr* were also downregulated in *Cpne3*-silenced cells. *Glut2* and *Gck* are key players in glucose uptake and glucose-sensing machinery in pancreatic β-cells to respond to physiological blood glucose changes^30,31^. The low affinity of GLUT2 permits glucose sensing and controls the circulation's glucose uptake rate. Several reports have shown that defects in glucose-sensing machinery impair insulin secretion, leading to severe hyperglycemia. The present study provides evidence that the decreased expression of GLUT2 was accompanied by reduced glucose uptake efficiency, which is a crucial driving factor for insulin release^32^.

Moreover, insulin signaling in β-cells is essential to maintain proper function^33^. The significant decrease in *Insr* after *Cpne3* silencing could lead to a defect in insulin secretion. Silencing of *Insr* in pancreatic β-cell has been shown previously to impair insulin secretion^33^. Collectively, these results indicate that *CPNE3* has a possible role in insulin sensing by reducing the expression of regulators of glucose sensing and insulin signaling.

Although the mechanism behind the role of *CPNE3* in insulin secretion is not clear. A speculated mechanism arises from the fact that *CPNE3* contains two Ca^2+^—and phospholipid-binding domains, ‘C2 domains’ which are found in protein such as protein kinase C (PKC), phospholipase C (PLC) and synaptotagmin^34^. PKC and PLC are well-known for their crucial role in regulating insulin secretion β-cells^27,35^. Synaptotagmins are thought to play roles in exocytosis and membrane trafficking phenomena^36^. Nevertheless, more functional investigations are warranted to dissect the exact mechanism through which *CPNE3* affects β-cell function.

In conclusion, our data suggest that *CPNE3* is a novel regulator in pancreatic β-cell function. Our data might pave the way to consider *CPNE3* as a drug target to develop a new molecular therapy for T2D.

## Materials and methods

### Microarray and RNA-sequencing from human pancreatic islets

Microarray expression data (GEO, accession number: GSE41762) from human pancreatic islets were retrieved from a publicly available database. Robust Multi-array Analysis methods were used to normalized the raw data as described previously^37^. RNA-seq data with accession number; GSE50398 were obtained from the GEO database. Data normalization was processed as previously described^38^.

### Culturing of INS-1 cell line and siRNA transfection

INS-1 (832/13) cells, a gift from Dr. C. Newgard from Duke University, were maintained in a complete RPMI 1640 medium as previously described^39^. Cells were seeded in a 24-well plate (200,000 cells/well) in Antibiotics free medium overnight. At confluency ~ 60%, cells were transfected with 40 nM of two siRNA sequences against CPNE3 (IDs: S162185 and S162183) (Thermo Fisher Scientific, USA) using 1.0 µl of lipofectamine 3000 transfection reagent in Opti-MEM media (Thermo Fisher) as previously described^39^. A previously defined negative control sequence was used at a final concentration of 40 nM^40^. After 24 h of transfection, the media was replaced with complete RPMI 1640 media with antibiotics and qPCR was performed to assess silencing efficiency.

### Insulin secretion assays

Glucose-stimulated insulin secretion (GSIS) assay was performed 48-h post-transfection. Briefly, cells were washed twice with secretion assay buffer (SAB), then normalized for 2 h.

Glucose stimulation was done by incubating them in 1 ml SAB (pH 7.2, 114 mM NaCl, 4.7 mM KCl, 1.2 mM KH_2_PO_4_, 1.16 mM MgSO_4_, 20 mM HEPES, 2.5 mM CaCl_2_, 25.5 mM NaHCO_3_ and 0.2% Bovine Serum Albumin) containing either 2.8 mM glucose plus secretagogue, either 16.7 mM glucose, 35 mM KCL or 10 mM α-ketoisocaproic acid (α-KIC) for 1 h as previously described^41^. Secreted Insulin was determined using a rat insulin ELISA kit (Mercodia, Sweden).

For measurements of insulin content, the total protein was extracted from transfected cells using an M-PER reagent (Thermo Fisher Scientific) and quantified by Pierce BCA protein assay kit (Thermo Fisher Scientific). Diluted protein (1:250) was then used to evaluated insulin content using a rat insulin ELISA kit (Mercodia, Sweden). Finally, insulin content was normalized to the total amount of protein.

### Quantitative-PCR

RNA extracted from transfected INS-1 cells was subjected for cDNA synthesis using a high-capacity cDNA Reverse Transcription Kit (Thermo Fisher). Silencing efficiency and expression of target genes were assessed by qPCR using TaqMan gene expression assays; *Glut2* (Rn00563565_m1), *Ins1* (Rn02121433_g1), *Ins2* (Rn01774648_g1), *Pdx1* (Rn00755591_m1), *Insr* (Rn00690703_m1), *Gck* (Rn00561265_m1) and Rat *Hprt1* (Rn01527840_m1) as endogenous control or SYBR Green gene expression analysis with the corresponding primers (Table 1). qPCR reactions were run in triplicate in 96-well plates using QuantStudio 3 qPCR system (Applied Biosystems, USA). Relative gene expression was determined by 2^−ΔΔCt^ method.

**Table 1:** Sequences of SYBR-Green PCR primers.

S. no.	Genes/symbol	Forward primers (5′–3′)	Reverse primers (5′–3′)
1	*Cpne1*	CTTTGTCGGACTGAACGTGTT	CCAGCTCAGGTGTCTTGTTGT
2	*Cpne2*	CTCAACCCGGCCTTCTCTAAG	CCCAGGAAGTCATGCTCATCC
3	*Cpne3*	GATGGCGTGATCACAGACCTT	GGCTTCCATTGTCACCGTCTA
4	*Cpne4*	CAACACAGAGTCAGAACCCTC	CCTTTGGTGGGTGAACATGGT
5	*Cpne5*	CACGTCCCTCCACTACATGAG	CAGGGCAGGGAACATCTTGTC
6	*Cpne6*	GAGGTTCTTCGCTCCTGTTCA	TCCATCCTCAGCATCGAACAC
7	*Cpne7*	GACGGATATGTCGGACACTCG	GTGGTGAGCGTAGGATTCCAT
8	*Cpne8*	AGCCCACTTCTCTCCACTACA	GAGCTTTGCACCGAAACCAAG
9	*Cpne9*	TGATGAAGACCCCAACTGTGC	GTGTGATGACAGGCGCAAAG
10	*Hprt*	TTGTGTCATCAGCGAAAGTGG	CACAGGACTAGAACGTCTGCT

### Western blot analysis

M-PER mammalian protein extraction reagent (Thermo Fisher), containing a protease inhibitor cocktail, was used to extract protein lysate from transfected cells (Thermo Fisher). SDS-PAGE was used to separate 40 μg of protein lysate, followed by blotting onto a nitrocellulose membrane (Bio-Rad, USA). The membrane was blocked using 5% skimmed milk in Tris-buffered saline containing 0.1% Tween 20 (TBST) for 1 h. Subsequently, blot was incubated overnight at 4 °C with primary antibodies against GLUT2 (Anti-rabbit; 1:1000, #ab54460, Abcam, UK), INSRβ (Antimouse; 1:1000; #ab69508, Abcam, UK), INSRα (Anti-rabbit; 1:1000; #ab5500, Abcam, UK), Insulin (Anti-mouse; 1:1000; #8138s, Cell signaling Technology, USA), GCK (Anti-rabbit; 1:500; #ab37796, Abcam, UK) PDX1 (Anti-rabbit; 1:3000, #ab47267, Abcam, UK), or β-actin (1:5000; #A5441, Sigma-Aldrich, Germany). The membrane was incubated with secondary antibodies (anti-mouse #7076S and antirabbit #7074S, Cell signaling Technology, USA) at 1:1000 dilutions at room temperature for 1 h. ECL substrate kit (Bio-Rad, USA) was used to detect chemiluminescence. Bio-Rad Image Lab software (Bio-Rad, USA) was used to detect protein bands. Quantification of the bands was done using Image J software. In all experiments, β-actin was used as an endogenous control (see Supplementary Information).

### Apoptosis assay

Twenty-four hours of post-transfection, a complete RPMI medium was used to culture the cells in the presence and absence of a mixture of pro-apoptotic cytokines (100 ng/ml IL-1β, 125 ng/ml TNFα, and 125 ng/ml IFNγ) for 24 h as previously described^42^. Briefly, buffer saline was used to wash cells, followed by resuspension in 500 μl of Annexin-V Binding Buffer (BD Biosciences, USA). Five μl of each annexin V-FITC and propidium iodide were used to stain the cells for 10 min in the dark, followed by analysis using BD FACS Aria III flow cytometer (Becton Dickinson, USA).

### Cell viability assay

A 20 × 10^4^ of transfected cells were seeded in a 96-wells plate, 48 h of post-transfection. Cells were incubated with 10 µl of MTT (5 mg/ml) solution (Sigma-Aldrich) and set for 2 h at 37 °C. Formazan crystals were then dissolved in DMSO, and absorbance at 570 nm was detected using a microplate reader. The average 570 nm absorbance values were used to calculate the percentage of cell viability based on the following formula: % cell viability = (OD 570 nm of sample/OD 570 nm of control) × 100.

### Glucose uptake assay

A fluorescent glucose analogue, 2-[*N*-(7-nitrobenz-2-oxa-1,3-diazol-4-yl) amino]-2-deoxy-glucose (2-NBDG) (Invitrogen #N13195), was used to measure glucose uptake in INS-1 cells according to the manufactory's instructions. Briefly, a 100 µM of 2-NBDG (dissolved in ethanol) was added over the transfected cells (as described above) (100 µM/1 ml medium/well) and incubated for one hour at 37 °C. Next, trypsin harvested cells were washed twice with cold-PBS, then centrifuged for 5 min at 1500 rpm at 4 °C. The supernatant was aspirated and cell suspended in 200 µl of cold-PBS in each tube. Cells were immediately analyzed and quantified by flow cytometry (BD FACS Aria™ III) at excitation 465 nm and emission at 540 nm .

### Cell proliferation assay (Edu labeling)

EdU Proliferation Kit (ab219801, abcam) was used to measure cell proliferation and conducted according to the manufacturer's instructions. In brief, transfected cell or control cells (2 × 10^5^ cells) were incubated with Edu for 4 h and then harvested. This was followed with fixation using 4% formaldehyde. After washing, EdU-incubated cells were labeled and analyzed using flow cytometry (BD FACS Aria™ III) (Supplementary Information).

### Statistical analysis

Differential expression analysis between diabetic and nondiabetic donors was analyzed by student t-test or nonparametric Mann–Whitney tests. Nonparametric Spearman's test was used for correlation analysis was done using. All statistical analyses were performed using GraphPad Prism (version 8.0.0 for Windows, GraphPad Software, San Diego, CA, USA, www.graphpad.com). Differences were considered significant at p < 0.05.

## Supplementary Information

Supplementary Figure 1.

Supplementary Figure 2.

Supplementary Figure 3.

Supplementary Figure 4.

Supplementary Figure 5.

Supplementary Figure 6.

Supplementary Legends.
