# Supplementary figures and images for: Copine 3 “CPNE3” is a novel regulator for insulin secretion and glucose uptake in pancreatic β-cells
Source: Sci Rep. 2021 Oct 19;11:20692. doi: 10.1038/s41598-021-00255-0 (PMC8526566; doi:10.1038/s41598-021-00255-0)

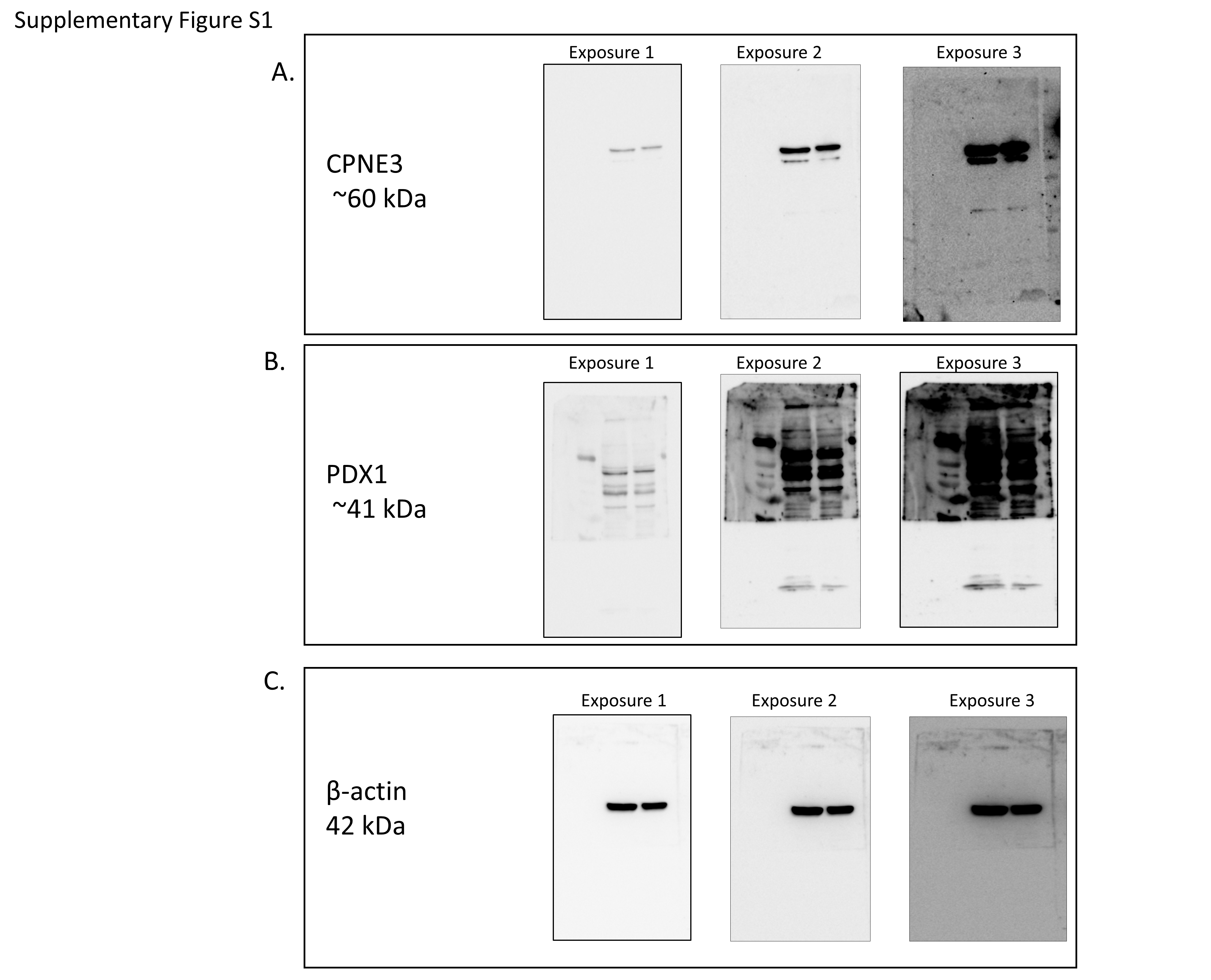

Supplement: Supplementary file 1 — Supplementary Figure 1. [file 41598_2021_255_MOESM1_ESM.tif]

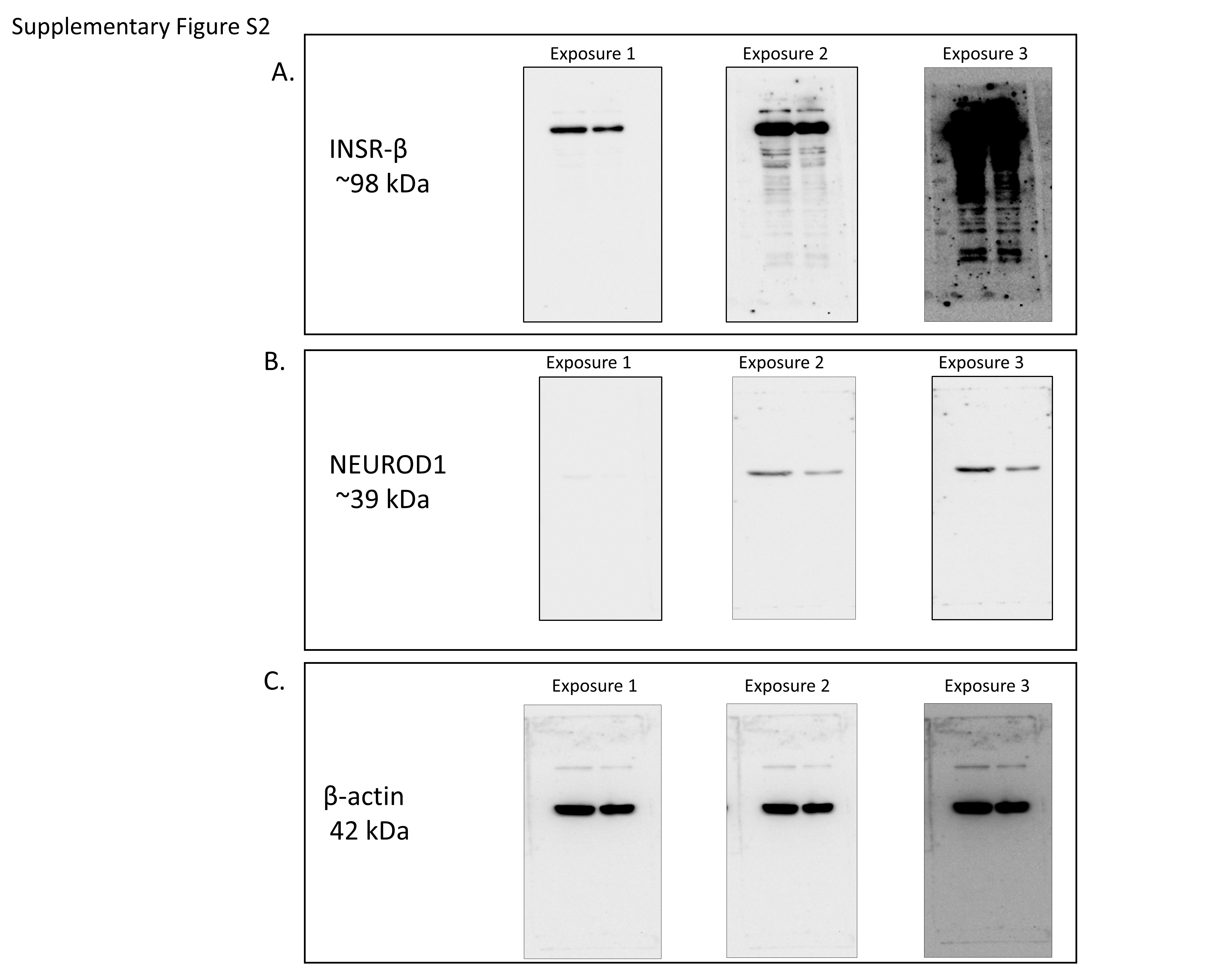

Supplement: Supplementary file 2 — Supplementary Figure 2. [file 41598_2021_255_MOESM2_ESM.tif]

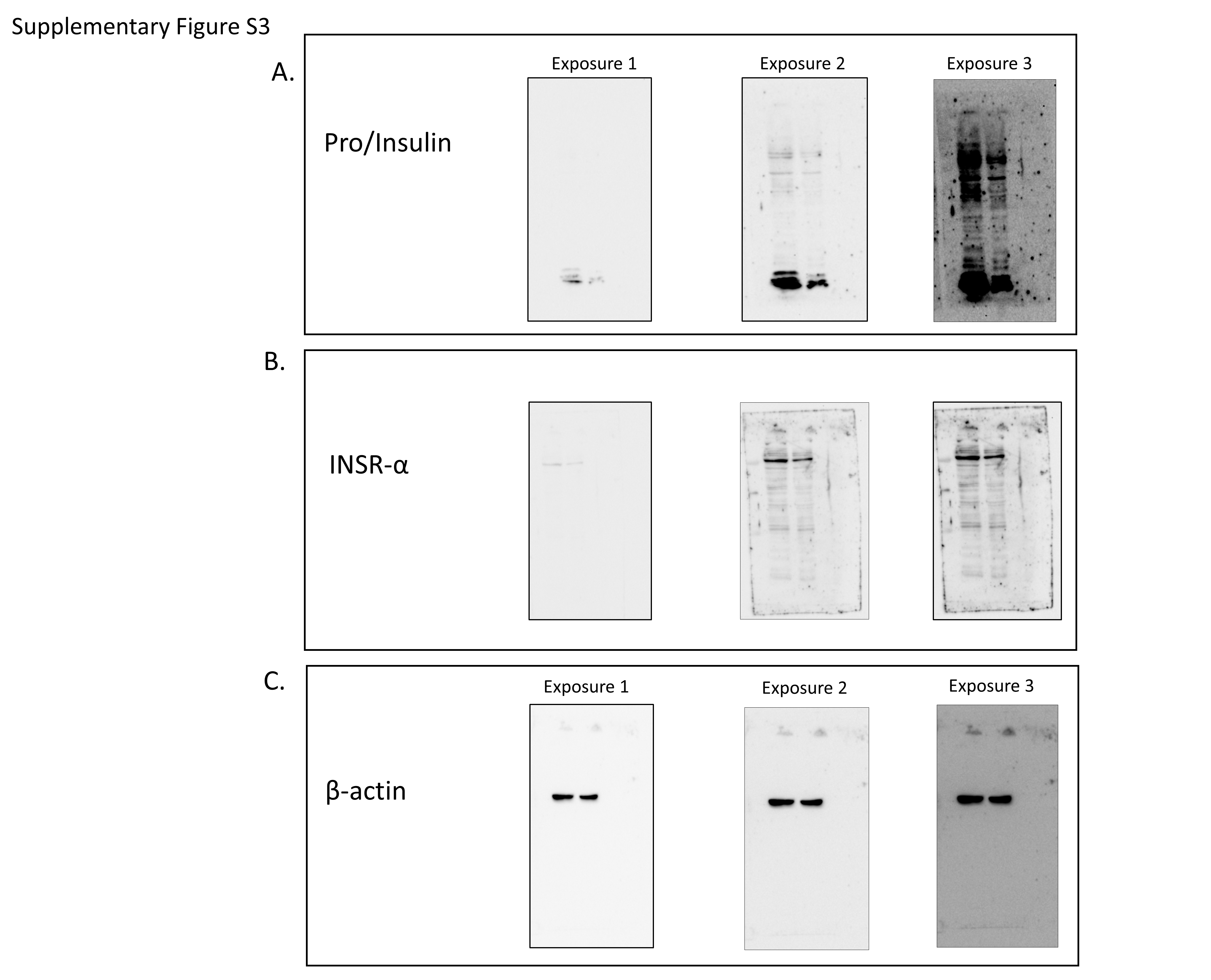

Supplement: Supplementary file 3 — Supplementary Figure 3. [file 41598_2021_255_MOESM3_ESM.tif]

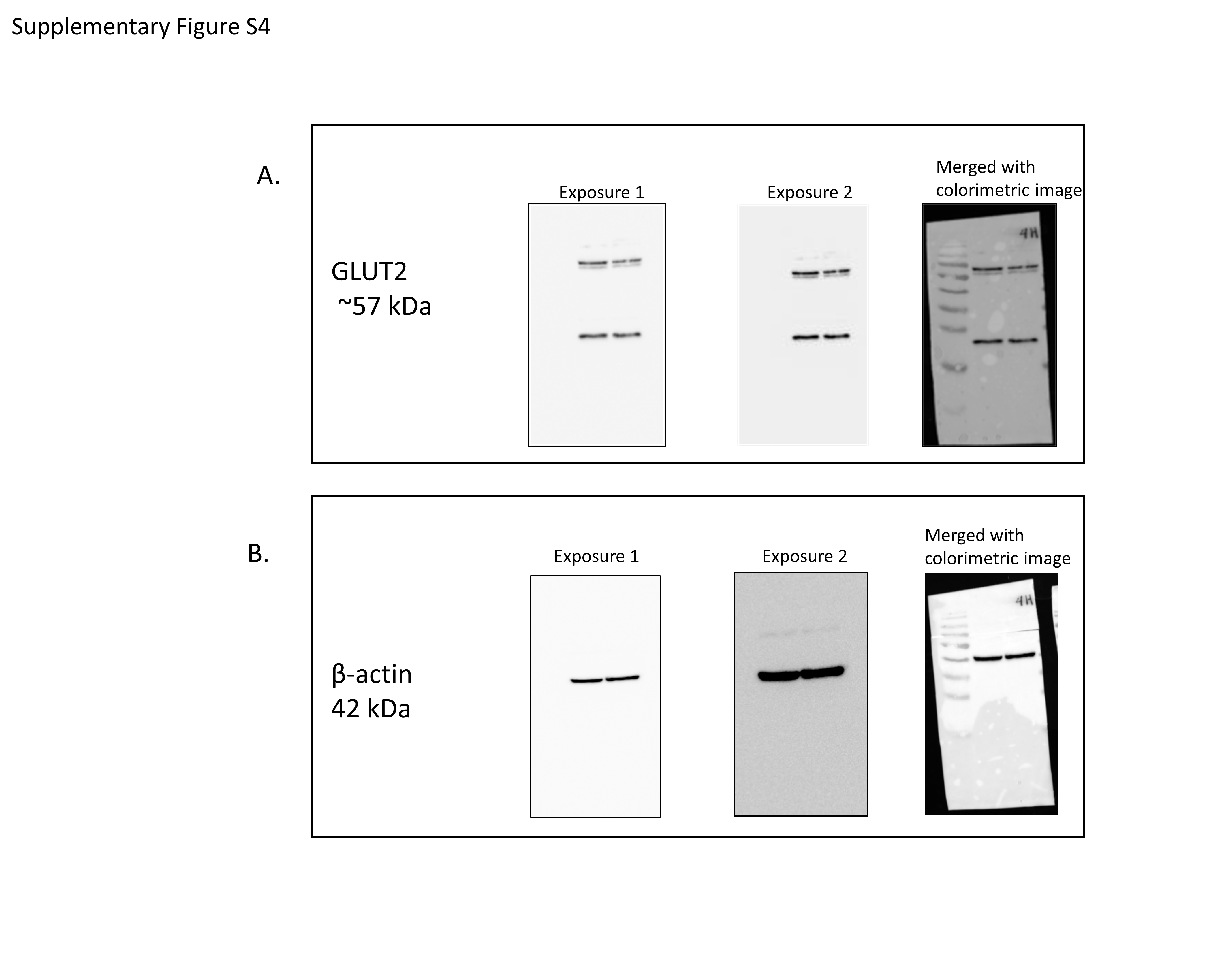

Supplement: Supplementary file 4 — Supplementary Figure 4. [file 41598_2021_255_MOESM4_ESM.tif]

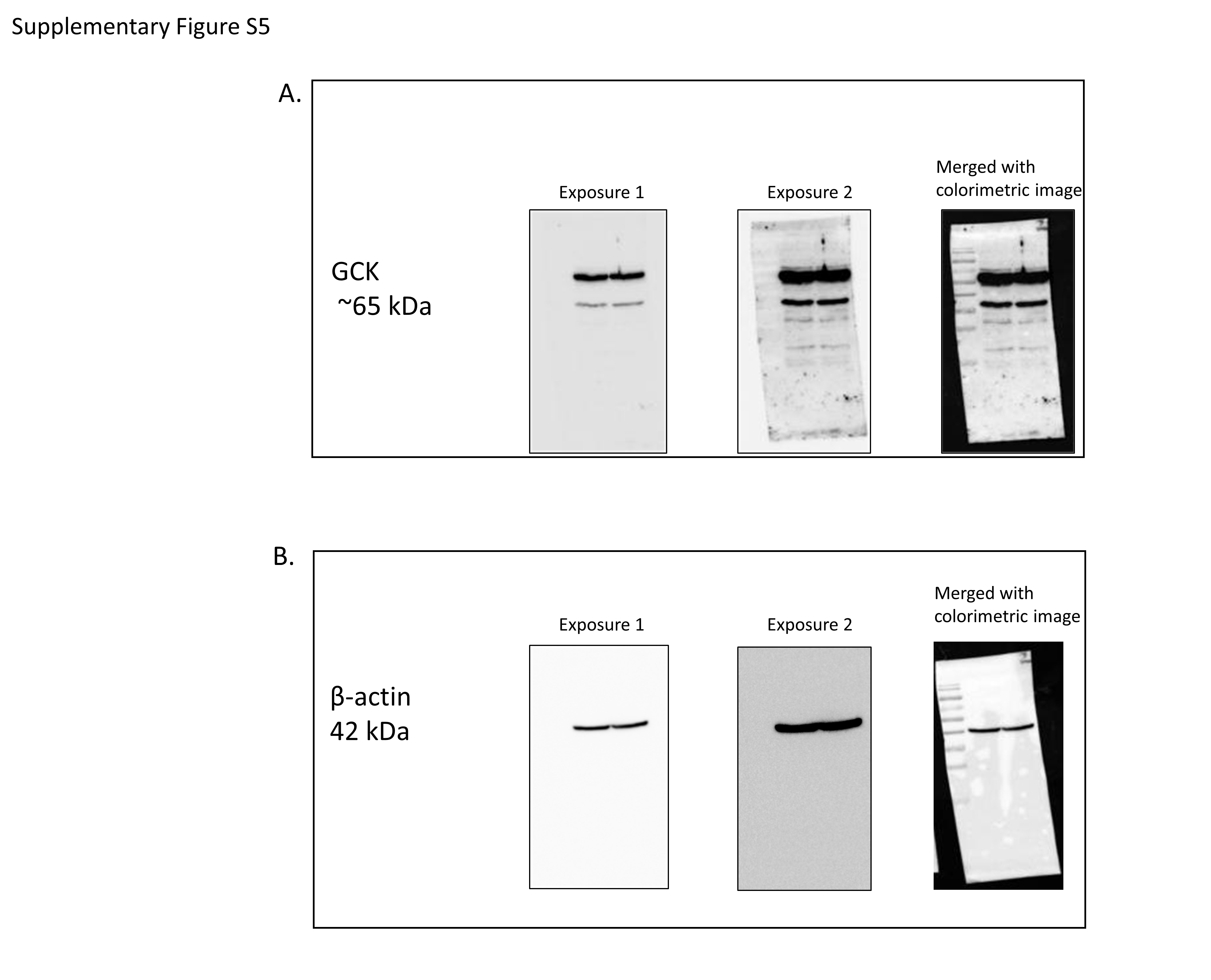

Supplement: Supplementary file 5 — Supplementary Figure 5. [file 41598_2021_255_MOESM5_ESM.tif]

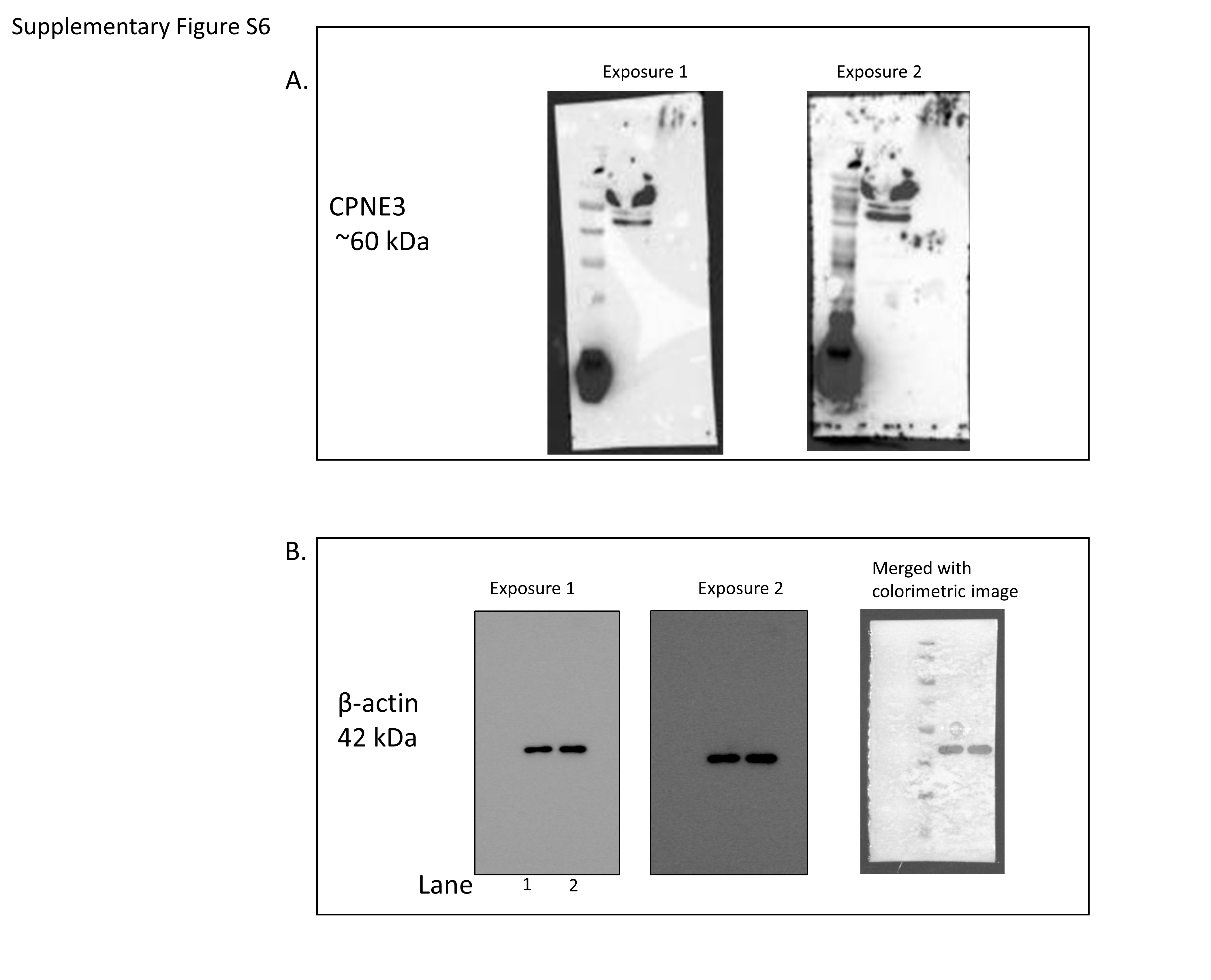

Supplement: Supplementary file 6 — Supplementary Figure 6. [file 41598_2021_255_MOESM6_ESM.tif]
