# Supplementary material for: Copine 3 “CPNE3” is a novel regulator for insulin secretion and glucose uptake in pancreatic β-cells
Source: Sci Rep. 2021 Oct 19;11:20692. doi: 10.1038/s41598-021-00255-0 (PMC8526566; doi:10.1038/s41598-021-00255-0)
Supplement: Supplementary file 7 — Supplementary Legends. [file 41598_2021_255_MOESM7_ESM.docx]

**Supplementary Figure S1.** (A) The full-length western blot expressions for CPNE3 in three different exposures. The cropped blot from exposure 2 was used as representative figure in the main article. Note: The membrane was cut at approximately 25 kDa after hybridizing with anti-CPNE3 antibody. The portion below 25 KDa was incubated with other antibody whereas the portion above 25 kDa was used hybridizing the anti-PDX1 antibody. (B) The full-length western blot expressions for PDX1 in three different exposures. The cropped blot from exposure 1 was used as representative figure in the main article. (C) The full-length western blot for β-actin, which was used a loading control. The cropped blot from exposure 1 was used as representative figure in the main article.

**Supplementary Figure S2.** (A) The full-length western blot expressions for INSR-β in three different exposures. The cropped blot from exposure 1 was used as representative figure in the main article. (B) The full-length western blot expressions for NEUROD1 in three different exposures. The cropped blot from exposure 3 was used as representative figure in the main article. (C) The full-length western blot for β-actin, which was used a loading control. The cropped blot from exposure 1 was used as representative figure in the main article.

**Supplementary Figure S3.** (A) The full-length western blot expressions for pro-insulin/insulin in three different exposures. The cropped blot from exposure 2 was used as representative figure in the main article. (B) The full-length western blot expressions for INSR-α in three different exposures. The cropped blot from exposure 2 was used as representative figure in the main article. (C) The full-length western blot for β-actin, which was used a loading control. The cropped blot from exposure 1 was used as representative figure in the main article.

**Supplementary Figure S4.** (A) The full-length western blot expressions for GLUT2 in three different exposures. The cropped blot from exposure 1 was used as representative figure in the main article. (B) The full-length western blot for β-actin, which was used a loading control. The cropped blot from exposure 1 was used as representative figure in the main article.

**Supplementary Figure S5.** (A) The full-length western blot expressions for GCK in three different exposures. The cropped blot from exposure 1 was used as representative figure in the main article. (B) The full-length western blot for β-actin, which was used a loading control. The cropped blot from exposure 1 was used as representative figure in the main article.

**Supplementary Figure S6.** (A) The full-length western blot expressions for CPNE3 in human islets in two different exposures. The cropped blot from exposure 1 was used as representative figure in the main article. (B) The full-length western blot for β-actin, which was used a loading control. The cropped blot from lane 2 of exposure 1 was used as representative figure in the main article. Note: For loading control, same amount of was used in separate blot.
